# Genetics in public health: Rarely explored

**DOI:** 10.4103/0971-6866.69326

**Published:** 2010

**Authors:** Y. B. Aswini, S. Varun

**Affiliations:** Department of Preventive and Community Dentistry, Teerthankar Mahaveer Dental college and Research Center, Delhi Road, Moradbad, Uttar Pradesh, India; 1 Department of Pedodontics and Preventive Dentistry, Teerthankar Mahaveer Dental college and Research Center, Delhi Road, Moradbad, Uttar Pradesh, India

**Keywords:** Barriers, community genetics, genetics, public health

## Abstract

The availability and the integration of genetic information into our understanding of normal and abnormal growth and development are driving important changes in health care. These changes have fostered the hope that the availability of genetic information will promote a better understanding of disease etiology and permit early, even pre-symptomatic diagnosis and preventive intervention to avoid disease onset. Hence, our aim was to review and provide the insight into the role of genetics in public health and its scope as well as barriers. The use of genetics along with their goals and essential public health functions are discussed. From the era of eugenics to the present era, this area has seen many turns in which geneticists have put through their effort to tie together the strings of both molecular genetics and public health. Though still the dark clouds of eugenics, the predictive power of genes, genetic reductionism, non-modifiable risk factors, individuals or populations, resource allocation, commercial imperative, discrimination and understanding and education are hanging above. The technological and scientific advances that have fundamentally changed our perception of human diseases fuel the expectations for this proactive health.

## Introduction

The mission of health professionals is to “fulfill society’s interest in assuring conditions in which people can be healthy”. This mission requires that we respond to ever-changing priorities and advancements in the scientific world. The past few decades have witnessed major technological advances. Breakthroughs in human genetics provide great promise for improving the health of the public. Genetics has been heralded by some as the new “revolution” in health care,[1–4] suggesting a rapid, fundamental change to, or overthrow of, existing health paradigms. Discoveries in genetics are already impacting society’s health in numerous ways. Every day, health professionals and the general public are provided information about exciting discoveries in areas such as cancer, heart disease, and birth defects, creating expectations for better health services.

Earlier, the science of human genetics was focused on micro-level health influences, and clinical genetics on rare, single gene disorders, providing diagnosis, risk estimation, reproductive options, and some newborn screening.[5] Present studies are clarifying previously unrecognized genetic and phenotypic heterogeneities and attempting to unravel the complex interactions between genes and environment by applying new statistical modeling approaches to twin and family data. Linkage studies using highly polymorphic DNA markers are providing a means of locating candidate genes, including quantitative trait loci (QTL). Apart from the host genome, considerations of the microbial genome have also impacted significantly on clinical practice.

In the decades to come, insights and techniques of molecular genetics will have great influence on prevention and health care. Health care providers should anticipate important new developments rather than just wait and see. For community doctors who can impossibly oversee all relevant developments in sufficient detail, close communication with the community and clinical genetic specialists is necessary to keep pace with the progress. With regard to genetic counseling and reproductive medicine, working agreements between primary care and specialist centers are important. Community-based genetic epidemiology has become a basic science in understanding the human genome.[6] Hence, this review aims to have some insight into the role of genetics in public health and its scope as well as barriers. A bullion search showed 85 related articles in which only 42 relevant articles were consulted. Apart from this, other sources like the book and Google search were obtained.

There has been a historic association between public health and genetics, beginning in the 19^th^ century, when public health and the eugenics movement shared common ground in values and ideas, programs, and personnel. In 1939, high profile geneticists were promoting improvement of the genetic constitution of the population through voluntary eugenics, facilitated by changing social conditions and human attitudes. Simultaneously, public health was focused on preventing disease primarily through the control of infection and malnutrition. There was an unspoken collaboration in place whereby public health could continue to prevent the deaths of the unfit so long as eugenics prevented the unfit from passing on their defects, thus counteracting “degeneration” of the population. In the 1940s, many people, including those who were involved with public health, withdrew their support for eugenics, as the ethics behind these practices were increasingly questioned. By the 1950s, human genetics had disentangled itself from eugenics and the practice of non-directive genetic counseling was introduced. This divergence was maintained throughout the second half of the 20^th^ century with public health shifting focus from infectious diseases to complex, chronic diseases and geneticists concentrated on mapping the human genome. Effective presentation of well-researched science and expression of realistic views should facilitate a more comfortable convergence in time.[7–14]

Clinical epidemiological methodology can contribute a lot to the quality of molecular clinico-genetic studies. Long-term follow-up to evaluate predictions and interventions needs more attention and can easily be integrated into primary care medicine. Primary health care providers, motivated to learn complex materials and new skills in order to assist their patient’s genetics, can do so in a relatively short time period.[15]

Professor Leo ten Kate rejects the term “public health genetics”, and proposes to use “community genetics” instead, because the latter refers to values that are not safe with the first. The term “public health genetics” has been introduced to denote the interface between genetics and public health, and is used in titles of training courses and names of research groups. It reflects attempts of the public health profession to cope with, and make best use of, the rapid advances in genetics. Genetic tests can, for example, be used in screening programs for disease or for targeting health promotion interventions. “Community genetics”, on the other hand, reflects attempts of clinical geneticists to apply their counseling methods to the whole population. According to ten Kate, clinical genetics embodies a non-directive approach that is badly needed when genetics is applied at a population-wide level. The public health approach is seen to be too directive, and to have an undue focus on achieving health gains for the population as a whole, instead of helping individuals to make autonomous choices, for example, when it comes to the reproductive options that they face when presented with genetic information.[16]

It is evident that genetics will become a fundamental component of the policy and practice roles of public health agencies by 2010,[17,18] making careful consideration of the framework and process for meeting this essential challenge. A larger challenge for state and local public health officials is setting standards for the role of genetics within the broad scope of core public health functions. Public health officials may be expected to provide criteria for 1) using genetic tests to predict the probability of disease and impact of interventions; 2) using genetic screening and services throughout the life span; and 3) preventing inappropriate uses of genetic testing.

## Three Core Public Health Functions and Genetics

The three core public health functions and genetics are discussed below.[19]

### 1) Assessment

To improve health, it is important to understand how genetics interacts with other factors. Therefore, it is necessary to regularly collect, analyze, and share information, including genetic information and environmental interactions related to health conditions, risks, and community resources. The surveillance is needed to determine:

- the population frequency of genetic variants that predispose people to specific diseases, both common and rare;
- the population frequency of morbidity and mortality associated with such diseases; and
- the prevalence and effects of environmental factors known to interact with the given genotypes in producing disease.

Establishing criteria for genetic testing recommendations may involve reassessing data using additional vital statistics or other factors. Other factors include the availability of quality genetic resources in the community, the appropriateness of genetic technologies offered to the community, the accessibility of clinical and genetic services, the costs and benefits of using genetic technology, and the community’s knowledge of the use of genetics to improve health. This information is necessary for state health officials and others responsible for providing health policy guidance, to enact policies and programs that are best for their communities.

### 2) Policy development

Public health policies also provide members of the public with objective guidance and information to empower them in decision making regarding the use of genetic technologies. Issues such as health insurance discrimination, population screening, and privacy and confidentiality require guidance from State Health Officials to ensure the public’s health and minimize potential harm.

### 3) Assurance

Agencies may collaborate with other public and private entities and educate public health staff and private health-care workers about the use of genetic information to improve health. Programmatically, the incorporation of up-to-date genetic information in areas such as maternal and child health, occupational health, and disease prevention programs will improve outcomes by providing better prevention information. This information should be available in formats that are appropriate to the target audience in terms of reading level and cultural competence. Enhancement of data systems to include genetic information, with appropriate privacy protections, can be part of ongoing considerations for program improvement. Outcome evaluations that include genetic information will create an opportunity to develop more effective policies and practices. Some health agencies may find it necessary to assure the availability and quality of laboratory and clinical genetic services in their state through licensing and certification activities.

## Ten Essential Public Health Services and Genetics

The ten essential services used[19,20] are given below to outline the integration of genetics into public health policy and practice, where appropriate, and to identify desired goals.

### 1) Monitor health status to identify community health problems

The development and maintenance of a strong health data collection system with the capacity to monitor genetic factors that affect health status and identify health problems is one of the essential public health services. Data collected in these systems could include genetic variants, health status, demographics, interventions, environmental triggers, and safety and efficacy of genetic technologies. They provide new insights into prevention.

#### Goals

1. Analyze incidence, mortality, and morbidity data to prevent and reduce the burden of disease and to associate the data with genetic predisposition and environmental triggers.
2. Identify opportunities for including genetic information in existing programs.
3. Develop data collection systems for genetics that can be integrated with existing data systems (e.g., birth defects registries, vital statistics, birth and death certificates, cancer registries, laboratory reporting).
4. Identify genetic information that is currently collected in existing data systems.
5. Identify communities that could benefit from genetic information and interventions.
6. Develop a system for analyzing the validity and utility of genetic tests.

### 2) Diagnose and investigate health problems and health hazards in the community

Applied public health research into the causes of health problems, including relevant genetic factors, is the key to understand how diseases can be prevented and to reduce their burden in the community.

#### Goals

1. Identify genetic risk factors to increase opportunities for early intervention, reduction of disease burden, and primary prevention of disease throughout the life span.
2. Identify environmental elements to which individuals may be particularly susceptible.
3. Develop a health promotion (social marketing) plan that empowers citizens to use genetic information appropriately to reduce their risk of disease.
4. Train personnel to assess genetic factors when investigating environmental health hazards and to create behavior change programs.

### 3) Inform, educate, and empower people about health issues

#### Goals

1. Inform the general public and policymakers about genetics and its impact on health.
2. Provide consistent information through a range of focused health education programs so that informed decisions regarding genetic health issues can be made.
3. Assess community needs for genetic information and services.

### 4) Mobilize community partnerships at the state and local levels to identify and solve health problems

#### Goals

1. Establish effective communication with community members regarding genetic issues.
2. Establish a committee of accountable community leaders with equal levels of participation in decision making to form genetic policies and practices.
3. Ensure the relevance of genetic policies and programs to the communities they are designed to serve and protect.

### 5) Develop policies and practices that support individual and community health efforts

#### Goals

1. Apply population-based genetic information to state policies and programs to improve individual and community health.
2. Develop a strategic plan to guide the integration of genetics into public health practice and policies.

### 6) Enforce laws and regulations that protect health and ensure safety

#### Goals

1. Develop legislation, statutes, and regulations that provide for the optimal use of genetic information to improve health, while protecting clients and consumers from the misuse of genetic information.
2. Provide leadership and guidance for public health genetic policies.

### 7) Link people to health services, including genetic services, and assure the provision of health care when otherwise unavailable

#### Goals

1. Create provisions for high-quality, culturally competent genetic services for those who need or desire them.
2. Ensure that high-quality, clinically valid genetic tests are available.
3. Develop genetic information and services that are culturally competent and effective in improving health.

### 8) Assure a public health and personal health care workforce competent in genetics

#### Goals

1. Create and maintain a public health workforce that is competent in public health genetics.
2. Provide opportunities for the current public health workforce to obtain continuing education in genetics.
3. Create opportunities for continuing education credit for all health professionals in genetics whenever possible.
4. Prepare current public health students to participate in programs that incorporate genetic information to promote health.

### 9) Evaluate effectiveness, accessibility, and quality of personal and population-based health services, including genetics

#### Goals

1. Assure the availability and accessibility of up-to-date genetic programs, services, tests, and treatments.
2. Conduct outcomes evaluation of available genetic services to determine their effectiveness.
3. Review and evaluate information related to the clinical utility and validity of genetic tests.

### 10) Research for new insights and innovative solutions to health problems

#### Goals

1. Identify and assess genetic research findings to determine the appropriateness of incorporating them into public health practices.
2. Assess the social, economic, and ethical impact of this information in determining its appropriateness for public health.
3. Ensure that genetic information is continually updated and incorporated into the public health infrastructure.

There are many barriers to effective convergence and approach to prevention and management of complex diseases from a multidisciplinary perspective. They are as follows.

### 1) Fear of eugenics

The main objective for using genetic information in public health today is not to enhance, change, or remove one’s genes, but to promote their optimal expression. Neither the possibility of a new eugenics era nor the changes in social values and economic influences can be ruled out.

### 2) The predictive power of genes

Not all genetic variants found to be associated with disease are clinically useful. Genetic tests should be introduced only when the predictive power is established within particular populations, and with knowledge of the “number needed to screen” (analogous to the “number needed to treat” concept) to prevent one case of disease Cost-benefit analyses and rigorous evaluation of genetic screening programs is essential.[21]

### 3) Genetic reductionism

Some public health professionals are concerned that at a time when there are moves to address inequalities in socioeconomic and political factors affecting health identification of genetic risk might move us back toward the “single cause for single disease” paradigm (analogous to the “germ theory” of the 19^th^–20^th^ century). A reductionist approach can be useful for identifying genetic associations with disease and elucidating etiological pathways in a research setting, but does not reflect the way genes operate in complex biological systems.

The term “individualistic fallacy” has been used to describe the situation where the major population determinants of health are ignored and the focus is on individual level variables – a criticism of genetic as well as other individual risk factor epidemiology.[22] The challenge that has been presented to epidemiologists is to embrace multiple levels of risk, from the molecular to the population and societal level, and new statistical techniques are being designed to integrate these levels of risk.

### 4) Non-modifiable risk factors

Public health evolved on the premise that genes could not be modified, effectively disqualifying them as targets for intervention. However, for complex diseases, genetic information may be used either pre-symptomatically or after the disease onset, for targeted interventions including diet, medication, and lifestyle modifications. Genetic information may motivate people to improve their health behavior, or, at the other extreme, it may lead to a fatalistic view of genetic risk with people shunning preventive behaviors or treatments.[23] The use of genetic information to improve risk identification may emphasize the high risk approach to public health.

### 5) Individuals or populations?

Public health, which seeks to improve the health of populations, has to recognize the importance of the individual. These changes offer challenges to public health practitioners and epidemiologists to encompass the individual view while maintaining efforts to promote the health of populations through a collective approach. This may be facilitated via informed consent and also the provision to opt out of public health programs, such as the one now in place for newborn screening.

### 6) Resource allocation

Imbalance in distribution of public resources in health care has been the cry of every public health professional.[24] A major area of concern is the prioritization of competitive research funding in favor of genetics. Another area of concern is the predicted investment in health services required for new genetic technologies and tests.[25] A symposium on community genetics in developing countries was held in India in 2002. An estimated half million babies are born every year in India with a birth defect, and birth defects have overtaken infection as a cause of perinatal mortality. Therefore, government support of existing, integrated local and district health centers and practitioners has been strongly advocated. The symposium discussed the establishment of community-controlled prenatal and newborn screening programs,[26] hand in hand with education and awareness campaigns. To seriously impact on health in developing countries through genomics, a global approach with “innovative financing mechanisms” is required.[25]

### 7) Commercial imperative

Commercial laboratories may bypass recommended pre-test genetic counseling when offering “over the counter” genetic testing. There are over 100 websites worldwide offering genetic testing for a variety of purposes, for example, parentage, identity, forensic, immigration, health-related genetic tests, and DNA banking. Significantly, 12 commercial organizations have been identified that offer adult genetic susceptibility testing. Any promotion of this type of testing will have ramifications for the public purse because of the increased need for follow-up health services. It may also exacerbate inequalities in access and there are concerns about privacy, safety, and quality. It is unclear how often patents for gene sequences related to susceptibility genes for complex conditions will be granted and then enforced, but they potentially have much greater public health implications.

### 8) Discrimination

Genetically susceptible population subgroups may be identified, marginalized, or discriminated against in various ways – the creation of a “genetic underclass”.[27] Family relationships, insurance (life, travel, and health), employment, finance, adoption, migration, forensic, and legal settings (paternity testing) are all examples of where genetic discrimination may occur. In reality, there have not been widespread cases of genetic discrimination yet,[27] but possibilities do exist. After many years of negotiation, the US Senate passed the Genetic Information Nondiscrimination Act of 2003 and it now remains to be seen if the House of Representatives will pass it. This bill would prevent health insurers and employers from using genetic information to determine eligibility, set premiums, or hire and fire people.[28]

### 9) Understanding and education

The complexity of genetics dictates the need for specialized languages and bodies of information. Genetic literacy assumes that the average person can evaluate the credibility of information that has implications for personal and public health, but most do not have this skill. Multidisciplinary education programs for health professionals are needed on the scientific, ethical, legal, and social issues related to public health genetics, as are programs on bioinformatics and statistical genetics, cultural anthropology, and health behavior.

A greater use of family history information to stratify individuals into average, moderate, and high risk for common diseases has been proposed, to guide prevention strategies. Those who are already identified as high risk based on family history are benefiting from predictive testing for single gene disorders (for example, some cancers), with those at moderate risk next in line. In time, testing might be broadened to those at average risk of common complex disorders. There is an urgent need for the integration of genetic screening into the public health services in India.[20]

It will take time but will clarify uncertainties and most importantly affirm that research and development in genetics need not diminish the importance of social and environmental determinants of health, and can in fact render the interventions more effective than before. The Massachusetts Department of Public Health (MDPH) has identified the following areas of focus for statewide public health genetics initiatives (2003–2005).[28]

### Professional education and training

- Promote genetic education and training for public health and health care professionals to assure awareness of emerging issues and appropriate utilization of new genetic technologies.
- Work in collaboration with statewide organizations, professional groups, and schools of public health and medicine to promote integration of genetics into professional practice.
- Promote established public health genetic competencies for health care and public health professionals.

### Public education

- Foster the public understanding of scientific developments in human genetics and associated ethical, legal, and social issues.
- Initiate and support collaborative public education and training programs that bridge knowledge gaps.

### Access to services

- Promote access to family-centered, culturally and linguistically appropriate genetic information and counseling, clinical and support services.
- Reduce financial, geographic, cultural, and linguistic barriers to access to genetic services and family support through coordination of services.
- Promote delivery of community-based genetic services and improve access to quality, cost-effective care.

### Information and referral

- Develop and maintain a statewide genetic resource database for public education, program planning, policy development, and quality assurance related to genetic services.
- Increase public health capacity for information, referral, and technical assistance.

### Data systems

- Develop integrated data systems to improve data coordination for public health service planning.
- Strengthen data collection efforts in collaboration with laboratories and comprehensive genetic centers to develop systems that monitor rates of genetic-related conditions and utilization of genetic technologies.

### Public policy

- Facilitate development and implementation of public policies pertaining to clinical, ethical, legal, and social aspects of genetic services.
- Maintain communication with professional organizations and foster compliance with clinical and laboratory standards related to genetic medicine, counseling, and education.

The first international conference on community genetics, which was held in Canada in June 2000 (Jonquière, Québec), offered the opportunity for common reflection and discussion on a definition of community genetics.[29] The real challenge now facing genetics is how to devise and deliver appropriate services so that individuals, families, and communities are sufficiently empowered and supported to make informed decisions about their genetic health. Community-based genetic counseling and education are the central components of the infrastructure that would deliver those services.

In view of the current developments, community practitioners must integrate community genetics into their daily routine and critically anticipate possibly relevant innovations. The convergence of public health and genetics holds the possibility of improved understanding of the etiology, prevention, and management of complex diseases such as diabetes, dementia, heart disease, cancer including oral cancers, dental diseases, and syndromes.

## References

[1] Collins FS, McKusick VA (2001). Implications of the human genome project for medical science. JAMA.

[2] Bell J (1998). The new genetics in clinical practice. BMJ.

[3] van Ommen GJ, Bakker E, den Dunnen JT (1999). The human genome project and the future of diagnostics, treatment and prevention. Lancet.

[4] Wallace H (2002). The need for independent scientific peer review of Biobank UK. Lancet.

[5] Pass K, Khoury M, Burke W, Thompson E (2000). Lessons learned from newborn screening for Phenylketonuria. Genetics and public health in the 21st century.

[6] Knottnerus JA (2003). Community genetics and community medicine. Fam Pract.

[7] Holtzman NA, Marteau TM (2000). Will genetics revolutionize medicine?. N Engl J Med.

[8] Venter JC, Adams MD, Myers EW, Li PW, Mural RJ, Sutton GG (2001). The sequence of the human genome. Science.

[9] Harrap SB, Petrou S (2001). Utility of genetic approaches to common cardiovascular diseases. Am J Physiol Heart Circ Physiol.

[10] Hart TC, Ferrell RE (2002). Genetic testing considerations for oral medicine. J Dent Educ.

[11] Halliday JL, Collins VR, Aitken MA, Richards MP, Olsson CA (2004). Genetics and public health-evolution, or revolution?. J Epidemiol Community Health.

[12] Paul DB, Spencer HG (1995). The hidden science of eugenics. Nature.

[13] McPherson JD, Marra M, Hillier L, Waterston RH, Chinwalla A, Wallis J (2001). A physical map of the human genome. Nature.

[14] Yoon PW, Chen B, Faucett A, Clyne M, Gwinn M, Lubin IM (2001). Public health impact of genetic tests at the end of the 20^th^ century. Genet Med.

[15] Kolb SE, Aguilar MC, Dinenberg M, Kaye CI (1999). Genetics education for primary care providers in community health settings. J Community Health.

[16] Mackenbach JP (2005). Community genetics or public health genetics?. J Epidemiol Community Health.

[17] Slavkin HC (2001). Human genome, implications for oral health and diseases, and dental education. J Dent Educ.

[18] Townsend GC, Aldred MJ, Bartold PM (1998). Genetic aspects of dental disorders. Aust Dent J.

[19] Framework for public health genetics policies and practices in state and local public health agencies. http://www.depts.washington.edu/corefeti/assess.htm-8/9/08.

[20] (2004). The Australian Public Health Genetics Consortium Genetics and Population Health Fremantle, Australia, August 8-10.

[21] Burke W, Atkins D, Gwinn M, Guttmacher A, Haddow J, Lau J (2002). Genetic test evaluation; information needs of clinicians, policy makers, and the public. Am J Epidemiol.

[22] Pearce N (2000). The ecological fallacy strikes back. J Epidemiol Community Health.

[23] Hunt K, Davison C, Emslie C, Ford G (2000). Are perceptions of a family history of heart disease related to health-related attitudes and behaviour. Health Educ Res.

[24] Leeder S Resource allocation and the genetic revolution?. http://www.onlineopinion.com.ao/view.asp?article=1162.

[25] Singer PA, Daar AS (2001). Harnessing genomics and biotechnology to improve global health equity. Science.

[26] Verma IC, Bijarnia S (2002). The burden of genetic disorders in India and a framework for community control. Community Genet.

[27] Rothenberg KH, Terry SF (2002). Before it’s too late-addressing fear of genetic information. Science.

[28] http://www.genome.gov/11510227.

[29] Brisson D (2000). Analysis and integration of definitions of community genetics. Community Genet.

